# Incobotulinum Toxin-A in Professional Musicians with Focal Task-Specific Dystonia: A Double Blind, Placebo Controlled, Cross-Over Study

**DOI:** 10.5334/tohm.903

**Published:** 2024-06-27

**Authors:** Steven J. Frucht, Mary Catherine George, Alexander Pantelyat, Eckart Altenmueller, Alexandra Nmashie, Jocelyn M. Jiao, Michael Chen, David Feng, Susan Shin, Michelle C. Kaku, David Simpson

**Affiliations:** 1 New York University Grossman School of Medicine, US; 2 Icahn School of Medicine at Mount Sinai, US; 3 Johns Hopkins School of Medicine, US; 4 Institute of Music Physiology and Musicians’ Medicine of the University of Music, Drama and Media, Hannover, DE; 5 Department of Pediatrics, New York Medical College, US; 6 Stanford University School of Medicine, US

**Keywords:** musician, dystonia, incobotulinum toxin, booster dose

## Abstract

**Background:**

Musician’s focal task-specific dystonia is a complex disorder of fine motor control, with incomplete understanding of its etiology. There have been relatively few trials of botulinum toxin in upper limb task-specific dystonia, and prior studies have yielded variable results, leading to skepticism regarding the utility of this approach in elite performers.

**Methods:**

We conducted a double-blind, placebo-controlled, randomized, cross-over study of incobotulinum toxin-A in 21 professional musicians with focal upper extremity task-specific dystonia affecting performance on their instrument, using a novel paradigm of initial injections followed by booster injections at two- and four-week intervals. The primary outcome measure was the change in blinded dystonia rating of the active arm by two expert raters using a Clinical Global Impression numeric scale at week 8 compared to enrollment.

**Findings:**

19 men and 2 women with musicians’ dystonia were enrolled over a six-year period. Nineteen patients completed the study. Analysis of the primary outcome measure in comparison to baseline revealed a change in dystonia severity of P = 0.04 and an improvement in overall musical performance of P = 0.027. No clinically significant weakness was observed, and neutralizing antibodies to toxin were not found.

**Interpretation:**

Despite its small sample size, our study demonstrated a statistically significant benefit of incobotulinum toxin-A injections as a treatment for musicians’ task-specific dystonia. Tailoring the use of toxin with booster injections allowed refinement of dosing strategy and outcomes, with benefits that were meaningful to patients clearly visible on videotaped evaluations. In addition to its application to musicians’ dystonia, this approach may have relevance to optimize application of botulinum toxin in other forms of focal dystonia such as blepharospasm, cervical dystonia, writer’s cramp, and spasmodic dysphonia.

## Introduction

Focal task-specific dystonia is an unusual and intriguing disorder of motor control. Affected patients develop dystonia, (involuntary movements producing sustained, twisting postures), in an affected body part specifically triggered by tasks such as writing, throwing a ball, swinging a golf club, or playing a musical instrument [1]. Musicians’ arm dystonia, a form of focal task-specific dystonia (FTSDma), typically affects professional musicians at the peak of their careers with mean age of onset of 36 years [2]. The development of hand or arm dystonia is a watershed event in the life of a performing artist, with devastating financial and psychological impact. FTSDma is not rare, with a lifetime incidence of 1 in 200 among professional musicians, and a prevalence measured in diverse populations of 2% of performing artists [3]. Both genetic and environmental factors are likely involved in the development of musicians’ dystonia, and the disorder has affected notable musicians such as Robert Schumann (piano) [4], Yehudi Menuhin (violin), Gary Graffman (piano), Leon Fleisher (piano), Peter Oundjian (violin) and Keith Emerson (piano).

The hand that sustains the greater technical burden appears to be preferentially affected in FTSDma—the right hand in pianists, left hand in string instrumentalists (violin, viola), and the right hand in plectrum instruments (guitar, banjo) [5]. Once present, symptoms of this painless disorder of motor control typically progress and worsen until affected musicians are unable to continue their careers. Adjacent fingers of the ulnar-innervated hand are most affected, and the pattern of involved muscles and specific triggers of dystonia vary significantly from patient to patient [5]. Men are affected four times as often as women, and rare families have been reported with familial musicians’ dystonia [6].

Treatments for FTSDma date back to the late nineteenth century. Oral medications (anticholinergics, baclofen), physical therapy, retraining protocols, constraint-induced movement therapy, sensory retuning protocols (e.g. Braille reading), and even stereotactic surgery (Voa thalamotomy) have been employed [7,8]. However, the most common approach to treatment relies on the use of injections of botulinum toxin (BoNT) to the affected arm or hand.

BoNT injections have revolutionized the treatment of many forms of focal dystonia, and level A evidence supports its use in blepharospasm, cervical dystonia, spasmodic dysphonia, and other forms of dystonia [9]. BoNT injected into muscles selectively blocks synaptic vesicle fusion by interfering with SNAP-25 function (for BoNT type A, the most used form) [10]. Injections are exceptionally well tolerated for these indications, with the most common side effect of transient minor weakness in the muscles injected. Patients with various forms of focal dystonia receive injections in three-to-four-month intervals, usually enjoying significant clinical benefit and improvement in quality of life [11]. In contrast, in a double-blind, randomized, placebo-controlled trial of BoNT injections for writer’s cramp, eighteen of twenty patients receiving active injections reported weakness in the arm, sometimes lasting more than three months, and only half decided to continue treatment [12]. A long-term follow-up study of BoNT injections for focal hand dystonia revealed that most patients treated in practice chose to discontinue treatment, usually due to insufficient benefit from treatment [13].

FTSDma presents significant therapeutic challenges unique to the disorder. In patients with blepharospasm, cervical dystonia, spasmodic dystonia or leg dystonia, the goal of treatment is to improve the severity of involuntary movements and functional performance. For musicians, the goal of treatment is to achieve a musically and professionally relevant improvement in instrumental performance, without causing functionally significant weakness. While a 70% improvement in severity of blepharospasm or torticollis may be acceptable to a patient and their treating physician, most musicians desire and expect a more robust response. Musicians with dystonia also face critical time pressure dictated by their profession and its limited acceptance of disability and injury. As stated by the late Leon Fleisher, “there is no disabled list in music”, and a musician who cannot perform faces time pressure to either return to active status or to give up (typically less than a year) [11].

Several prior studies have evaluated the use of BoNT in musicians’ arm dystonia [14]. These studies share several limitations, and many questions remain unanswered, such as: what is the optimal method for injection?; which muscles and doses should be selected for each instrument and how to optimize performance?; and how should outcomes be measured? Available rating scales for musicians’ dystonia incompletely capture the phenomenologic complexity seen in clinical practice. This situation presents a formidable therapeutic challenge for the treating clinician caring for these often-desperate patients as well as for researchers investigating these conditions.

We aimed to address some of these challenges in the present study. We designed a double-blind, placebo-controlled, crossover study of Incobotulinumtoxin A (Inco-BoNT-A) injection vs. placebo for musicians’ arm dystonia (FTSDma). To assess patient outcomes, we videotaped patients performing musical excerpts that demonstrated their dystonia at each trial visit, and then randomized and blinded the videos, so they could be rated completely without bias. Two expert raters (EA, AP) scored both musical performance and dystonia severity using a seven-point CGI scale referenced to baseline performance of each patient before enrollment. In this manner, active vs. placebo status, and ordering of injections were blinded, removing therapeutic and timing bias. We employed a novel strategy of injections given at enrollment, and at weeks two and four in the active arm (and the placebo arm), to allow optimization of dosing patterns to account for the individual complexity of each patients’ dystonic pattern while attempting to preserve the blind. Similar injections of saline were employed in the placebo arm. We chose to use Inco-BoNT-A because this formulation has the lowest amount of accompanying chaperone proteins, and theoretically carries the lowest risk of engendering antibody formation in treated patients. While neutralizing antibodies have not been observed in prior trials of BoNT for FTSDma, many expert injectors raise concern that booster injections could generate neutralizing antibodies.

## Clinical Trial Design and Methods

**Figure 1:**
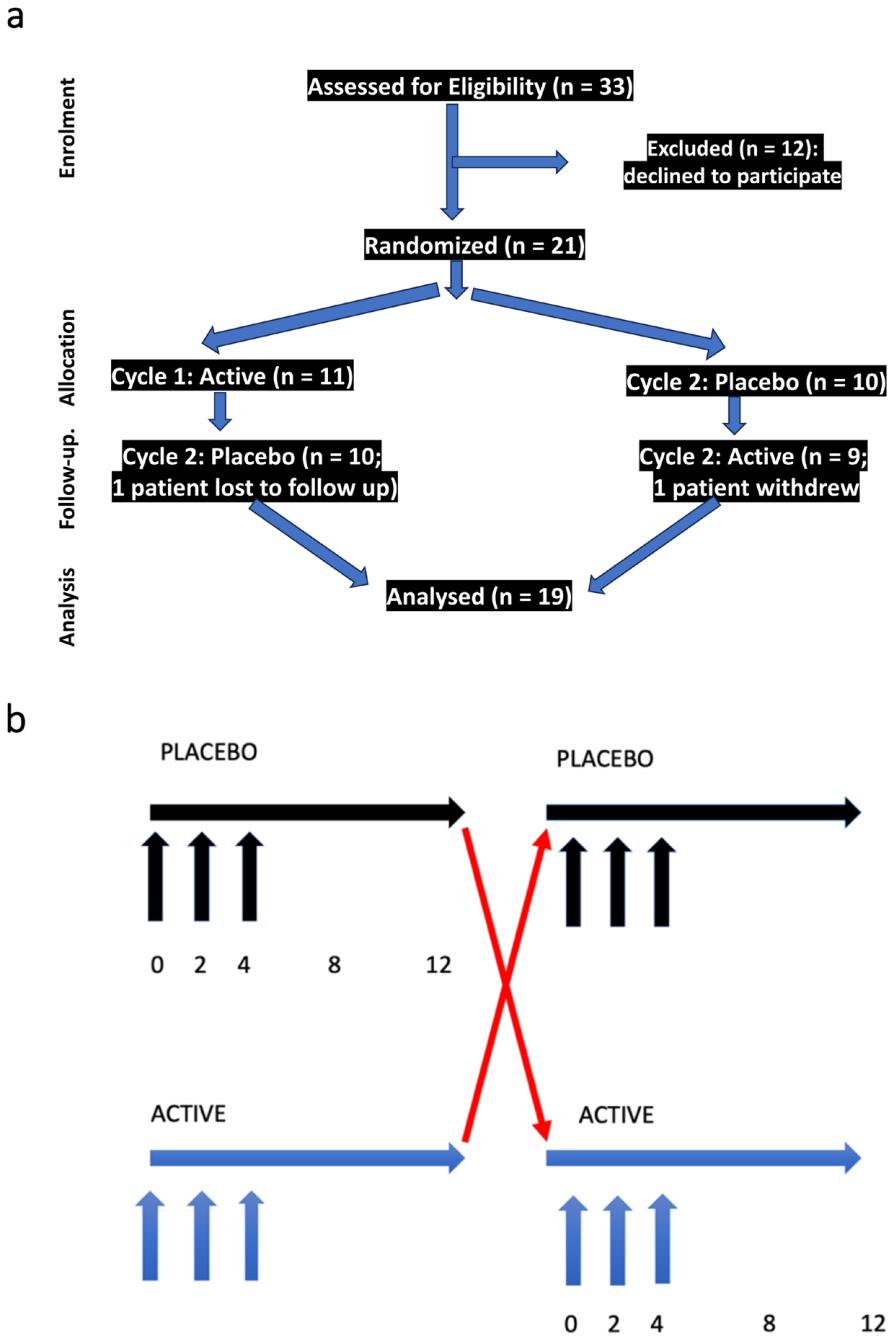
Trial Design. Figure 1 summarizes the trial design. Figure 1a summarizes patient assessments by CONSORT criteria. 33 patients were assessed for eligibility, and 12 declined to participate (due to the possibility of receiving placebo). 21 patients were randomized in double blind fashion, 11 to active arm first followed by placebo, 10 to placebo arm first followed by active. One patient was lost to follow up and one patient withdrew). 19 patients completed both placebo and active arms, and all were included in the analysis. Figure 1b further illustrates the trial design. Patients were randomly assigned in double blind fashion to either active or placebo arm. Visits occurred at weeks 0 (enrollment), 2, 4, 8 and 12 in each segment (active or placebo). Injections were performed at weeks 0, 2 and 4 of both active and placebo segments.

The clinical trial was approved by the institutional review boards of the Icahn School of Medicine at Mount Sinai Medical Center and NYU Langone Medical Center, registered at Clinicaltrials.gov (NCT02107261), and supported by UL1TR004419 from the National Center for Advancing Translational Science, National Institutes of Health. We conducted a double-blind, placebo-controlled, randomized, cross-over study of inco-BoNT-A vs placebo in two groups of patients: Group A--patients naïve to BoNT treatment, and Group B--patients with prior BoNT. Previous treatment with any BoNT product for any condition occurred ≥ 12 weeks prior to baseline enrollment. Trial design is summarized in Figure 1. All patients were professional musicians with focal upper limb task-specific dystonia affecting performance on their instrument, including seven pianists, six plectrum players (guitar, banjo), three drummers, two flautists, and one trumpeter, saxophonist, and violinist.

Patients were examined and evaluated for initial Inco-BoNT-A injection by the investigators (SF, DS), and again at weeks 2 and 4. Inco-BoNT-A or placebo was administered at the initial visit, and then at week 2 and week 4 at the discretion of the patient and injector (DS) based on their response and performance on their instrument. The patients were first evaluated by one investigator (SF), videotaped during performance, and then evaluated and injected by the other investigator (DS). Injections were performed using electrical stimulation to confirm targeting and avoid inadvertent injection of other muscles. Given the complexity and variability of each patient’s dystonic phenotype and triggers, patients were asked to play (while videotaped) for at least five minutes, and to specifically choose at least two representative passages, one particularly difficult (triggering the dystonia), and one that was easier. Patients were asked to repeat the two selected passages at each visit, to capture differences in performance of the same musical segments over time.

Each investigator (SF, DS) independently selected a pattern of injection and dosing of injection based on their evaluation at each visit. The investigators discussed the pattern and dosing recommendations at each visit in real time, until a consensus of the best approach was reached. Evaluation at week 8 was chosen as the point of maximal benefit and time of primary outcome assessment. Cycle 2 began at week 12 with similar cross-over visit schedules and repeat injections of either Inco-BoNT-A or placebo. Patients were evaluated at each visit with videotaping during performance, MRC rating, dynamometry, and subjective patient assessment by a visual analogue scale (VAS) selecting a point on a line from 0 (best musical performance) to 100 (worst musical performance). Blood specimens were drawn at baseline and at the final visit to test for the generation of neutralizing antibodies to Inco-BoNT-A, and to assess the risk of the potential for antibody generation in the booster injection approach.

After completion of all trial visits, video segments from each visit were randomized to order using a standard randomization table. Two independent expert raters (AP, EA) were given score sheets using a seven-point CGI scale and asked to rate both dystonia severity and musical performance for each video segment, compared to a reference tape of the patient at enrollment. The reference segment of video was purposely edited so that the rater would not recognize the video as baseline. The CGI scale constrained the raters to pick one of seven ratings compared to the reference segment: 0 (identical); +1 (minimal improvement); +2 (moderate improvement); +3 (marked improvement); –1 (minimal worsening); –2 (moderate worsening); –3 (marked worsening).

The primary outcome measure was the change in blinded dystonia rating score at week 8 compared to enrollment in the active arm. Secondary outcome measures included blinded musical performance scores, patient-rated questionnaires, motor strength testing utilizing the Medical Research Council (MRC) scale, dynamometry of the finger/wrist/elbow flexors to document any treatment-induced weakness, and patient Visual Analogue Scale (VAS) ratings of overall dystonia severity.

## Results

### Subject enrollment

Twenty-one patients (19 males, 2 females) with FTSDma were randomized consisting of Group A: 13 patients Naïve to BoNT, and Group B: 8 patients with prior BoNT treatment. Nineteen patients completed both cycles; 2 male participants discontinued after cycle 1 after changing their mind and have been excluded from the analysis. The study population’s ethnic demographics included 16 Caucasian, 2 African American and 1 Asian, with mean age of 50.2 (SD 11.6; range 26.9–68.4)). Clinical features of the twenty-one patients are summarized in **Supplementary tables 1 and 2**.

### Clinical Protocol

Eleven patients were randomized to placebo in cycle 1 and active drug in cycle 2 (*P*→*A*); ten patients were randomized to receive active drug in cycle 1 and then placebo in cycle 2 (*A*→*P*). Compliance with visits was excellent. Within the active arm, the mean dose of Inco-BoNT-A injected at first dose was 22.8U (7.5–45.0U). Nine patients received boosters at week 2 of the active arm (mean dose 16.7, range 5–35U), and ten patients received boosters at week 4 of the active arm (mean dose 8.5U, range 0.0–30.0) **(Supplementary tables 3 and 4)**. The decision to receive a booster at each visit was made independent of their prior treatment, focusing only on the residual dystonia and the need to improve their performance. Patients received boosters in the placebo arm as well based on the impression of the injector and examiner. Median total dose in the *P*→*A* arm was 115 units, and in the *A*→*P* arm 81.3 units, due to an outlier effect of one patient (a Rock drummer) who received higher doses. Bloods were drawn from 21 patients prior to enrollment, and from 18 patients at the completion of the study. Testing for neutralizing antibodies to Inco-BoNT-A was performed by Toxogen GmBH Lab, Hanover, Germany using the guidelines specified by https://pubmed.ncbi.nlm.nih.gov/9294406/.

### Outcomes

**Figure 2:**
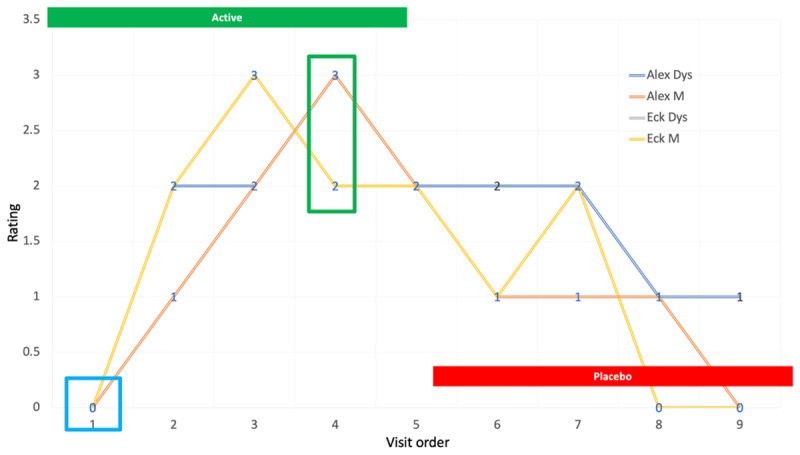
Graphic representation of ratings for a tabla player. Blinded CGI ratings of musical (M) performance and dystonia severity (D) for a table player are depicted in Figure 2. After completion of the trial, it was determined that the patient had been assigned to the active arm first. Video segments from each visit appear in the accompanying video.

The primary outcome measure was the change in blinded dystonia rating at week 8 in the active arm compared to baseline. P value was based on the Cochran-Mantel-Haenszal test using score = ridit option. The primary outcome measure for cycle 1 week 8 in comparison to baseline video rating for dystonia severity was P = 0.04, and rating for overall musical performance was P = 0.027. Examples of blinded ratings of a tabla player (a traditional Indian drum) with focal dystonia involving his right index finger appear in Figure 2.

Secondary outcome measures included patient-rated questionnaires, motor strength testing utilizing the Medical Research Council (MRC) scale, dynamometry of the finger/wrist/elbow flexors to document any treatment-induced weakness, and patient Visual Analogue Scale of overall dystonia severity. Patient VAS ratings of dystonia severity in the active arm were compared to ratings at enrollment. Two participants were excluded as outliers. One participant reported pain during week 8 visit and a 2^nd^ participant was delayed in returning for the study week 8 visit. The mean VAS in the active arm at baseline was –20.4 (SE6.47) and the mean in the placebo arm was –11.33 (SE6.47). Patient-rated questionnaires (Perceived Stress and Modified Hospital Anxiety and Depression Scale) performed at active week 8 compared to ratings at baseline did not show significance in either test. We modified the HADS to use language that was related to the context of being a musician. Motor strength testing using MRC at week 8 active vs. enrollment were unchanged. Participants scored normal strength. There were 34 dynamometry measures available in the study of the 21 participants, but only sixteen had complete measures for all the muscles. There was significant reduction from baseline rating for 11 measures in active arm compared to placebo group, demonstrating the change in strength in pinch for digits 2, 3 and 4, grip strength, finger flexors digit 2 and 3 and pronation. Levels of neutralizing antibodies at enrollment and completion of the study revealed no evidence of antibody generation. We also did not observe clinically significant evidence of lack of response to toxin, although we did not separately test tasks unrelated to musical performance, relying instead on the patients’ report. Change in blinded dystonia ratings and musical performance ratings at week 8 compared to baseline appear in **Supplemental Table 6**. Data from secondary outcomes are available online in **Supplemental tables 7–11**. An illustrative video and legend are available online in supplement form.

## Adverse events

No serious adverse events occurred during the study. Three patients reported minor transient discomfort at the injection site lasting less than 48 hours. Clinically noticeable weakness was insignificant.

## Video

Example of a video of instrumental performance from a patient with FTSDma is reviewed in the accompanying video and legend (supplemental data available online).

## Discussion

In this double-blind, placebo-controlled, crossover trial of Inco-BoNT-A injection using booster injections, musicians affected by task-specific dystonia experienced a significant improvement in both dystonia severity and musical performance. Injections were well tolerated, with transient treatment-induced weakness as the only significant adverse event. Resistance to toxin as demonstrated by clinically significant lack of benefit or the development of neutralizing antibodies was not seen.

Our trial design and analysis possessed significant strengths. To our knowledge, only one other double-blind trial of BoNT in musician’s dystonia has been reported, with smaller sample size and results that were exploratory [15]. In addition to blinding of the enrolling investigator, injector and subject to active vs. placebo arm, and by employing two expert raters blinded to active vs. placebo injections and order of visits, we believe that we have set a high bar for establishing efficacy. Despite the relatively small sample size, both ratings of dystonia severity and musical performance showed significant overall improvement. Although not specifically studied, allowing booster injections at weeks two and four appeared to optimize treatment response. Specifically, by using low starting doses and slowly escalating booster doses, we were able to avoid clinically bothersome treatment-induced weakness while obtaining meaningful benefit. After the completion of this trial, we have continued to treat patients with musicians’ dystonia using this approach and have found it to be very helpful in optimizing functional outcomes for these patients.

We are aware of certain limitations in our trial design and performance. Half of the subjects enrolled had received BoNT injections in the past, potentially impacting their response to treatment. We injected patients who played a variety of instruments, rather than focusing on one instrument, to better reflect the challenges of clinical practice. Both points reflect the rarity of musician’s dystonia in practice, and more than five years were needed to complete recruitment in this single-center study. We chose a two-week window for boosters, and empirically limited boosters to weeks two and four. Other strategies could have been employed, however this one proved to be effective.

When deciding on trial design in 2012, we assumed (from experience injecting patients with other forms of focal dystonia) that a three-month interval was sufficient for the effects of toxin to have worn off. We were surprised to observe a significant carry-over effect of toxin in patients who received active injections first. In subsequent experience injecting patients with musicians’ dystonia in clinical practice over the last decade, we and others have observed that the response of musicians’ dystonia to injections often lasts more than three months, sometimes as long as six months. This carry-over effect, while unintended, did present an unexpected limitation to the crossover design. We believe that our choice of primary endpoint of dystonia rating at the point of maximal benefit from the active arm vs. baseline at enrollment helped mitigate this effect. While the investigators (and some patients) were able to guess correctly that they were receiving active toxin, they persevered and did not drop out from the study when crossed over to the placebo arm, a testament to their dedication to the study and to the aim of establishing this therapy for their indication. Finally, we chose not to employ one of the standard rating scales that have been used in studies of musicians’ dystonia [16]. These scales in our view are insensitive to the variety of phenotypes of dystonia affecting various instrumentalists and patients. Our choice of a CGI scale constrained our two expert blinded raters to similar, somewhat arbitrary ratings. However, by employing two expert raters (both movement disorder experts and performing musicians) and blinding ratings to both active vs. placebo arm and trial order, we believe that our design more than makes up for these limitations.

Musicians affected by task-specific dystonia face challenges not seen in other patients with focal dystonia. FTSDma affects the critical activity that defines their personal and professional identity. The late Leon Fleisher, one of the most prominent musicians affected with FTSDma, described it in the following way: “*when the gods strike, they know where to hit you*” (Fleisher). Treatment for FTSDma also faces a higher bar than treatments for blepharospasm, torticollis and writer’s cramp. Unless a treatment improves functional performance, is well tolerated, and exerts its effect reasonably quickly, it will be of little use to performing artists who depend on playing to work. Many questions face the clinician embarking on treating FTSDma with BoNT, including: what are the primary dystonic movements (flexion vs. extension)?; which parts of the hand or arm are affected?; which movements are compensatory, and should therefore be avoided?; which muscles are driving the dystonia?; how sensitive will the patient be to injections? Without a booster paradigm, clinical experience suggests that it is extremely difficult to optimize treatment to adequately address these issues. We believe that the success of our trial depended in large part on the effective use of booster injections.

FTSDma offers a unique window into disorders of elite motor performance. Listening carefully to patients’ descriptions and observing their performance in real time (and later by video after unblinding), several lessons emerged from the study. While not specifically included as outcome measures, we believe that it may be useful to describe these lessons for the interested reader. Flexion of the fingers and wrist occurs much more commonly than extension in affected musicians. Isolating individual fascicles of the flexor digitorum superficialis and profundus for injection is critically important for achieving benefit without weakening the ability of the neighboring finger to perform. We were impressed with the involvement of the lumbricals in many patients, particularly keyboard and woodwind instrumentalists. Maintaining the “bridge” of the hand (the MCP joint positioned in mild flexion) allows the distal fingertips to be optimally positioned to depress the keys of a piano or clarinet for example. Particularly in woodwind instrumentalists, overactivity of a lumbrical flexes the finger at the MCP join and extends the distal finger, pushing it off the key. Virtually all instruments involving the hand require contact of the distal fingertip with the instrument, often with sub-millimeter precision. Injection of toxin into the flexor digitorum profundus must therefore balance the need to address distal finger flexion with the knowledge that weakness in this muscle may imperil the ability of the finger to adequately engage with the instrument.

We believe that careful targeting of the intended muscles for BoNT injection is critical in maximizing benefit and minimizing inadvertent injection of neighboring muscles. While we often use the combination of electrical stimulation and ultrasound targeting in our clinical practice, we chose to simplify the procedure in the trial, using only electrical stimulation guidance, since this is routinely performed in our hospital. Alternatively, other clinicians may prefer ultrasound guidance as the most favorable technique in future studies.

Finally, we observed that musicians affected by dystonic phenotypes that were simpler (one or two adjacent fingers in flexion, dystonia of mild severity) experienced more benefit from treatment than those with complex dystonic patterns involving multiple muscle groups and joints. In the last two decades, musicians with dystonia are likely seeking treatment earlier in their disease course due to increased awareness of the disorder and social media, allowing many to benefit from carefully applied injections.

Clinically relevant weakness was rare in this trial, and dynamometer measurements documented only mild reduction in strength. Nevertheless, improvement in dystonia was significant and often obvious to the patient and investigator. This dichotomy between improvement in dystonia despite only mild treatment-induced weakness has been observed in other forms of dystonia treated with BoNT [17]. Various explanations have been proposed, including action of toxin on gamma muscle spindle afferents, producing benefit by emulating a prolonged sensory trick [18]. In this trial, and in clinical practice, we have observed that the improvements with injections in musicians often last beyond the usual three-month injection window, allowing performing artists to space their injections at four- to six-month or even longer intervals.

## Conclusion

We believe that the placebo-controlled, double-blind, crossover design, with further blinded ratings of randomized ordered videos by two expert raters, sets a high standard for efficacy. Despite the small sample size, this preliminary study demonstrated statistically significant efficacy of Inco-BoNT-A injections in FTSDma. Patients were offered treatment on conclusion of the trial in open label fashion, and the booster paradigm clearly allowed individualized optimization of muscle and dose selection. This trial suggests that tailoring use of Inco-BoNT-A to fit specific needs of elite performers yields clinically meaningful results and may inform future larger trials of Inco-BoNT-A in FTSDma and other forms of focal dystonia.

## Additional Files

The additional files for this article can be found as follows:

10.5334/tohm.903.s1 Supplemental Data. Tables 1–11.

10.5334/tohm.903.s2 Supplemental Video. Representative video clips from a participant in the trial are presented. The patient developed musicians’ dystonia affecting the right index finger while playing the Indian table. He was randomly assigned to the active arm first, receiving Inco-BoNT-A injections at visits 1, 2 and 3. After blinding was revealed, he received the following doses in active arm: visit 1—7.5 units to FDS digit II, 5 units to FDP digit II; visit 2: 5 units to FDS digit II; visit 3: 5 units of FDS digit II. At visit 4, 8 weeks after enrollment, he reported that his playing felt completely normal. On crossover to the placebo arm at visit 5, benefit from Bo-NT injections persisted in videos 6, 7 and 8, finally returning to baseline at visit 9 six months after initial enrollment. He has continued to receive periodic injections with excellent benefit after completing the trial.

## References

[1] Stahl CM, Frucht SJ. Focal task specific dystonia: a review and update. J Neurol. 2017; 264: 1536–41. DOI: 10.1007/s00415-016-8373-z 28039522 PMC5502053

[2] Frucht SJ. Focal task-specific dystonia of the musicians’ hand—a practical approach for the clinician. J Hand Ther. 2009; 22: 136–42. DOI: 10.1016/j.jht.2008.11.006 19272752

[3] Altenmuller E, Jabusch HC. Focal dystonia in musicians: phenomenology, pathophysiology, triggering factors, and treatment. Med Probl Perform Art. 2010; 25: 3–9. DOI: 10.21091/mppa.2010.1002 20795373

[4] Garcia de Yebenes J. Did Robert Schumann have dystonia? Mov Disord. 1995; 10: 413–7. DOI: 10.1002/mds.870100402 7565819

[5] Conti AM, Pullman S, Frucht SJ. The hand that has forgotten its cunning—lessons from musicians’ hand dystonia. Mov Disord. 2008; 23: 1398–406. DOI: 10.1002/mds.21976 18398917

[6] Schmidt A, Jabusch HC, Altenmuller E, et al. Dominantly transmitted focal dystonia in families of patients with musician’s cramp. Neurology. 2006; 67: 691–3. DOI: 10.1212/01.wnl.0000230148.00035.f9 16924027

[7] Enke AM, Poskey GA. Neuromuscualr re-education programs for musicians with focal hand dystonia: a systematic review. Med Probl Perform Art. 2018; 33: 137–45. DOI: 10.21091/mppa.2018.2014 29868689

[8] Horisawa S, Ochiai T, Goto S, et al. Safety and long-term efficacy of ventro-oral thalamotomy for focal hand dystonia: a retrospective study of 171 patients. J Neurology. 2019; 92: e371–77. DOI: 10.1212/WNL.0000000000006818 PMC6345121 30587520

[9] Dressler D, Altavista MC, Altenmuller E, et al. Consensus guidelines for botulinum toxin therapy: general algorithms and dosing tables for dystonia and spasticity. J Neural Transm. 2021; 128: 321–35. DOI: 10.1007/s00702-021-02312-4 33635442 PMC7969540

[10] Raman S, Yamamoto Y, Suzuki Y, Matsuka Y. Mechanism and clinical use of botulinum neurotoxin in head and facial region. J Prosthodont Res. 2023. DOI: 10.2186/jpr.JPR_D_22_00238 36740263

[11] Fleisher L, Midgette A. My nine lives: a memoir of many careers in music. Anchor Books New York. 2011.

[12] Kruisdijk JJM, Koelman JHTM, Obgerboer de Visser BW, et al. Botulinum toxin for writer’s cramp: a randomized, placebo-controlled trial and 1-year follow-up. J Neurol Neurosurg Psychiatry. 2007; 78: 264–270. DOI: 10.1136/jnnp.2005.083170 17185301 PMC2117645

[13] Lungu C, Karp BI, Alter K, et al. Long term follow-up of botulinum toxin therapy for focal hand dystonia: outcome at 10 or more years. Mov Disord. 2011; 26: 750–753. DOI: 10.1002/mds.23504 21506157 PMC3081109

[14] Zakin E, Simpson DM. Botulinum toxin therapy in writer’s cramp and musicians’ dystonia toxin. 2021; 13: 988. DOI: 10.3390/toxins13120899 PMC8708945 34941736

[15] Cole R, Hallett M, Cohen LG. Double-blind trial of botulinum toxin for treatment of focal hand dystonia. Mov Disord. 1995; 10: 466–71. DOI: 10.1002/mds.870100411 7565828

[16] Peterson DA, Berque P, Jabusch HC et al. Rating scales for musician’s dystonia: the state of the art. Neurology. 2013; 81: 589–98. DOI: 10.1212/WNL.0b013e31829e6f72 23884039 PMC3775681

[17] Rajan R, Srivastava AK, Anandapadmanabhan R, et al. Assessment of botulinum neurotoxin injection for dystonic hand tremor: a randomized clinical trial. JAMA Neurol. 2021; 78: 302–11. DOI: 10.1001/jamaneurol.2020.4766 33346814 PMC7754081

[18] Giladi N. The mechanism of action of botulinum toxin type A in focal dystonia is most probably through its dual effect on efferent (motor) and afferent pathways at the injected stie. J Neurol Sci. 1997; 152: 132–5. DOI: 10.1016/s0022-510x(97)00151-2 9415532

