# Supplementary material for: Incobotulinum Toxin-A in Professional Musicians with Focal Task-Specific Dystonia: A Double Blind, Placebo Controlled, Cross-Over Study
Source: Tremor Other Hyperkinet Mov (N Y). 2024 Jun 27;14:32. doi: 10.5334/tohm.903 (PMC11212776; doi:10.5334/tohm.903)
Supplement: Supplemental Data. — Tables 1–11. [file tohm-14-1-903-s1.pdf]

Supplemental Data (available online)

Table 1: Patient disposition and assignment to Active or Placebo arms

	P→A (N=11)	A→P (N=10)	Total (N=21)
Randomized	11 (100%)	10 (100%)	21 (100%)
Completed	10 (90.91%)	9 (90%)	19 (90.48%)
Discontinued	1 (9,09%)	1 (10%)	2 (9.52%)
Discontinued from cycle 1	0	0	0
Discontinued from cycle 2	1 (9,09%)	1 (10%)	2 (9.52%)
A: Active drug; P: Placebo. Note: A→P: Received the active drug injection in cycle 1 and placebo in cycle 2. P→A: Received the placebo in cycle 1 and active drug injection in cycle 2.			

Table 2: Demographic and Baseline Characteristics of Patients

Variable	P→A (N=11)	A→P (N=10)	Total (N=21)
Age (Years)			
N (miss)	11	10	21
Mean (SD)	52.3 (11.47)	47.9 (12.00)	50.2 (11.64)
Median	55.8	44.7	52.6
Min, Max	26.9 - 68.4	33.5 - 67.4	26.9 - 68.4
Weight (Kg)			
N (miss)	10 (1)	10 (0)	20 (0)
Mean (SD)	86.9 (14.16)	81.5 (7.62)	84.2 (11.41)
Median	82.8	81.4	82.1
Min - Max	67.0 - 118.0	65.3 - 89.2	65.3 - 118.0
Race, n (%)			
Asian	1 (9.1%)	0	1 (4.8%)
Black	0	2 (20.0%)	2 (9.5%)
White	10 (90.9%)	8 (80.0%)	18 (85.7%)
Gender, n (%)			
Female	1 (9.1%)	1 (10.0%)	2 (9.5%)
Male	10 (90.9%)	9 (90.0%)	19 (90.5%)
Ethnicity, n(%)			
Hispanic	1 (9.1%)	0	1 (4.8%)
Non-Hispanic	10 (90.9%)	10 (100.0%)	20 (95.2%)
Conmed, n(%)			
Yes	6 (54.5%)	5 (50.0%)	11 (52.4%)
No	4 (36.4%)	5 (50.0%)	9 (42.9%)
Missing	1 (9.1%)	0	1 (4.8%)

A: Active drug; P: Placebo. Note: A→P: Received the active drug injection in cycle 1 and placebo in cycle 2. P→A: Received the placebo in cycle 1 and active drug injection in cycle

Table 3: Study drug injection (Overall)

	P→A (N=11)	A→P (N=10)	Total (N=21)
**Total Inco-BoNT-A dose (U)**			
Number of patients	11	10	21
Mean (SD)	254.8 (380.5)	77.8 (36.03)	170.5 (284.9)
Median	115.0	81.3	87.5

Min - Max	25.0 – 129.0	22.5 - 135.0	22.5 - 1290
Per Injection:			
Number of patients	11	10	21
Mean (SD)	27.5 (25.15)	18.6 (10.96)	23.2 (19.77)
Median	19.0	20.0	19.0
Min - Max	7.0 - 82.0	4.0 - 42.5	4.0 - 82.0

Median total dose in the *P→A* arm was 115 units, and in the *A→P* arm 81.3 units, due to an outlier effect of one patient (a Rock drummer) who received higher doses.

Table 4: Cumulative dose per muscle and mean cumulative dose per muscle in placebo and active arms.

		Placebo		Active	
**Muscle**	**Number of Subjects**	***Cumulative dose per muscle per subject range**	**Mean cumulative dose**	***Cumulative dose per muscle per subject range**	**Mean cumulative dose**
Adductor Pollicis	1	0	0	2.5u	2.5u
Anterior Deltoid	1	150u	150u	125u	125u
EDC D2	2	10 – 25u	17.5u	0 – 5u	2.5u
EDC D3	1	10u	10u	15u	15u
EIP	3	15 – 25u	20u	5u	5u
FCR	1	110u	110u	50u	50u
FCU	2	32.5 – 90u	61.3u	37.5 – 60u	48.75u
FDI	1	0	0	2.5u	2.5u
FDP D2	2	5 – 12.5u	8.8u	5 – 15u	10u
FDP D3	3	7.5 – 17.5u	11.7u	5 – 20u	14.2u
FDP D4	3	10 – 25u	17.5u	2.5 – 17.5u	10u
FDP D5	3	7.5 – 17.5u	11.7u	0 – 15u	5.8u
FDS D2	5	15 – 35u	21.5u	0 – 22.5u	13.5u
FDS D3	8	12.5 – 35u	25.6u	12.5 – 32.5u	25u
FDS D4	10	10 – 45u	22.8u	0 – 35u	19.3u
FDS D5	8	5 – 35u	21.3u	0 – 40u	21.9u
FPB	1	5u	5u	2.5u	2.5u
Levator Scapulae	1	0	0	100u	100u
Lumbrical D2	4	0 – 7.5u	3.8u	2.5 – 10u	5.6u
Lumbrical D3	5	0 – 12.5u	4.5u	5 – 15u	9u
Lumbrical D4	5	7.5 – 35u	16.5u	5 – 17.5u	11.5u
Lumbrical D5	4	10 – 15u	12.5u	7.5 – 17.5u	13u
Pectoralis Major	1	180u	180u	160u	160u
Teres Major	1	120u	120u	90u	90u
Trapezius	1	0	0	100u	100u
Triceps Medial Head	1	60u	60u	50u	50u
Triceps Lateral Head	1	120u	120u	105u	105u

EDC: extensor digitorum communis; EIP: extensor indicis proprius; FCR: flexor carpi radialis; FCU: flexor carpi ulnaris; FDI: first dorsal interosseous; FDP: flexor digitorum profundus; FDS: flexor digitorum superficialis; FPB: flexor policis brevis.

Table 5: Study drug injection by week

param	Visit	Stat	P→A (N=11)	A→P (N=10)	Total (N=21)
Total dose (U)	Baseline visit (week 0)	n	11	10	21
Total dose (U)	Baseline visit (week 0)	Mean (SD)	45.0 (66.82)	22.8 (10.37)	34.4 (49.10)
Total dose (U)	Baseline visit (week 0)	Median	20.0	20.0	20.0
Total dose (U)	Baseline visit (week 0)	Min - Max	5.0 - 220.0	7.5 - 45.0	5.0 - 220.0
Total dose (U)	Week 2 booster	n	10	9	19
Total dose (U)	Week 2 booster	Mean (SD)	49.5 (76.66)	16.7 (8.75)	33.9 (57.06)
Total dose (U)	Week 2 booster	Median	20.0	15.0	17.5
Total dose (U)	Week 2 booster	Min - Max	7.5 - 260.0	5.0 - 35.0	5.0 - 260.0
Total dose (U)	Week 4 booster	n	11	10	21
Total dose (U)	Week 4 booster	Mean (SD)	49.5 (73.68)	8.5 (9.52)	30.0 (56.54)
Total dose (U)	Week 4 booster	Median	20.0	5.0	10.0
Total dose (U)	Week 4 booster	Min - Max	0.0 - 250.0	0.0 - 30.0	0.0 - 250.0
Total dose (U)	Crossover visit (week 12)	n	10	8	18
Total dose (U)	Crossover visit (week 12)	Mean (SD)	51.3 (76.90)	14.7 (8.91)	35.0 (59.27)
Total dose (U)	Crossover visit (week 12)	Median	18.8	15.0	16.3
Total dose (U)	Crossover visit (week 12)	Min - Max	10.0 - 250.0	5.0 - 25.0	5.0 - 250.0
Total dose (U)	Week 14 booster	n	10	7	17
Total dose (U)	Week 14 booster	Mean (SD)	38.0 (48.77)	15.0 (7.22)	28.5 (38.65)
Total dose (U)	Week 14 booster	Median	22.5	15.0	20.0
Total dose (U)	Week 14 booster	Min - Max	5.0 - 160.0	5.0 - 27.5	5.0 - 160.0
Total dose (U)	Week 16 booster	n	9	6	15
Total dose (U)	Week 16 booster	Mean (SD)	41.7 (50.54)	15.4 (8.28)	31.2 (40.76)
Total dose (U)	Week 16 booster	Median	17.5	16.3	17.5
Total dose (U)	Week 16 booster	Min - Max	5.0 - 150.0	5.0 - 25.0	5.0 - 150.0
Injection	Baseline visit (week 0)	n	11	9	20

Injection	Baseline visit (week 0)	Mean (SD)	4.7 (4.08)	3.9 (2.09)	4.4 (3.28)
Injection	Baseline visit (week 0)	Median	3.0	4.0	3.5
Injection	Baseline visit (week 0)	Min - Max	1.0 - 13.0	2.0 - 9.0	1.0 - 13.0
Injection	Week 2 booster	n	10	9	19
Injection	Week 2 booster	Mean (SD)	5.3 (3.74)	3.6 (1.81)	4.5 (3.04)
Injection	Week 2 booster	Median	4.0	4.0	4.0
Injection	Week 2 booster	Min - Max	1.0 - 13.0	1.0 - 7.0	1.0 - 13.0
Injection	Week 4 booster	n	11	10	21
Injection	Week 4 booster	Mean (SD)	5.4 (3.96)	4.6 (6.62)	5.0 (5.26)
Injection	Week 4 booster	Median	4.0	2.0	3.0
Injection	Week 4 booster	Min - Max	2.0 - 13.0	0.0 - 22.5	0.0 - 22.5
Injection	Crossover visit (week 12)	n	10	8	18
Injection	Crossover visit (week 12)	Mean (SD)	4.5 (4.28)	3.1 (1.25)	3.9 (3.29)
Injection	Crossover visit (week 12)	Median	2.0	4.0	3.0
Injection	Crossover visit (week 12)	Min - Max	1.0 - 13.0	1.0 - 4.0	1.0 - 13.0
Injection	Week 14 booster	n	10	8	18
Injection	Week 14 booster	Mean (SD)	4.7 (4.55)	2.8 (1.16)	3.8 (3.54)
Injection	Week 14 booster	Median	2.5	2.5	2.5
Injection	Week 14 booster	Min - Max	1.0 - 13.0	1.0 - 4.0	1.0 - 13.0
Injection	Week 16 booster	n	9	8	17
Injection	Week 16 booster	Mean (SD)	5.1 (6.01)	3.3 (1.04)	4.2 (4.41)
Injection	Week 16 booster	Median	2.0	4.0	3.0
Injection	Week 16 booster	Min - Max	1.0 - 17.0	2.0 - 4.0	1.0 - 17.0

Table 6: Change in blinded dystonia rating and musical performance rating at week 8 (active arm) compared to baseline for all participants:

Patient #	EA: Baseline Mus	EA: Week 8 Mus	AP: Baseline Mus	AP: Week 8 Mus	EA: Baseline Dys	EA: Week 8 Dys	AP: Baseline Dys	AP: Week 8 Dya
**1**	0	2	0	3	0	2	0	3
**2**	0	1	0	2	0	2	0	3
**3**	0	2	0	1	0	2	0	2
**4**	0	1	0	3	0	1	0	3
**5**	0	1	0	1	0	1	0	2
**6**	0	1	0	2	0	1	0	2
**7**	0	1	0	2	0	1	0	2
**8**	1	2	0	1	0	2	0	2
**9**	0	1	0	0	0	2	0	1
**10**	0	2	0	2	0	2	0	2
**11**	0	1	0	1	0	1	0	1
**12**	0	1	0	1	0	1	0	2
**13**	0	1	0	1	0	1	0	1
**14**	0	1	0	1	0	1	0	1
**15**	0	0	0	2	0	2	0	2
**16**	0	1	0	0	0	1	0	1
**17**	0	-2	0	1	0	-2	0	1
**18**	1	-1	0	0	1	-1	0	-1
**19**	0	0	0	0	1	0	1	-2

Table 7: Primary Efficacy endpoint: Dynamometer measures at the eight-week mark in the active arm compared to the baseline rating.

Method 1: Assumed that the injection effect is no more than 8 weeks, so the visit 1(day 0) and visit 5(week 12, the start of 2nd injection cycle) were considered baseline visit. Note: There were 34 dynamometer measures, among the 21 patients, only 16 patients had available rating data. There was significant reduction from baseline rating for 11 measures in active arm compared to placebo group.

Endpoint		XEOMIN	Placebo	Difference	P-value
Change from baseline in DYN_pnch_5_Avg at week 8 (Change: post-baseline - baseline)	LSMeans (SE)	-0.235(0.4102)	0.549(0.4111)	0.784(0.3675)	0.044
	95% CI	-1.084(0.613)	(-0.302,1.399)	0.024(1.544)	
Change from baseline in DYN_pnch_4_max at week 8	LSMeans (SE)	-1.948(0.5087)	0.536(0.5118)	2.484(0.5831)	<0.0001
	95% CI	-3(-0.895)	(-0.523,1.595)	1.278(3.69)	
Change from baseline in DYN_pnch_4_Avg at week 8	LSMeans (SE)	-1.415(0.4771)	0.648(0.4797)	2.063(0.5132)	0.001
	95% CI	-2.402(-0.428)	(-0.345,1.64)	1.001(3.124)	
Change from baseline in DYN_pnch_3_max at week 8	LSMeans (SE)	-1.767(0.6536)	0.793(0.6551)	2.56(0.68)	0.001
	95% CI	-3.119(-0.415)	(-0.562,2.148)	1.154(3.967)	
Change from baseline in DYN_pnch_3_Avg at week 8	LSMeans (SE)	-1.377(0.5814)	1.17(0.5824)	2.547(0.6298)	0.001
	95% CI	-2.579(-0.174)	(-0.034,2.375)	1.244(3.85)	
Change from baseline in DYN_pnch_2_Avg at week 8	LSMeans (SE)	-1.596(0.691)	0.371(0.6935)	1.966(0.8675)	0.033
	95% CI	-3.025(-0.166)	(-1.064,1.805)	0.172(3.761)	
Change from baseline in Grip_max at week 8	LSMeans (SE)	-16.153(5.0562)	2.93(5.0709)	19.083(4.5297)	<0.0001
	95% CI	-26.613(-5.694)	(-7.561,13.42)	9.712(28.453)	
Change from baseline in Grip_avg at week 8	LSMeans (SE)	-14.699(4.9926)	2.681(5.007)	17.381(5.5336)	0.005
	95% CI	-25.027(-4.371)	(-7.676,13.039)	5.934(28.828)	
Change from baseline in FF D2-3 max at week 8	LSMeans (SE)	-2.737(3.1312)	8.78(3.3806)	11.517(4.5418)	0.019
	95% CI	-9.249(3.774)	(1.75,15.81)	2.072(20.963)	
Change from baseline in pronation maximum pounds of force at week 8	LSMeans (SE)	-1.502(0.7331)	1.046(0.7328)	2.547(0.831)	0.005
	95% CI	-3.018(0.015)	(-0.47,2.562)	0.828(4.267)	
Change from baseline in pronation average pounds of force of 8 tries at week 8	LSMeans (SE)	-1.481(0.67)	1.198(0.6692)	2.679(0.7462)	0.002
	95% CI	-2.867(-0.095)	(-0.187,2.582)	1.135(4.222)	

Method 2: For the AP group (took XEOMIN injection in cycle 1), after comparing the difference between visit 1 (day 0) and visit 5(week 12, the start of 2nd injection cycle) for all measures **via paired t-test or paired Wilcoxon sign test** (if normal assumptions are significantly violated), **there were 7 measures (DYN_pnch_4_max, DYN_pnch_4_avg, DYN_pnch_3_max, DYN_pnch_3_avg, DYN_pnch_2_max, DYN_pnch_2_avg, Elbox_Flex_avg) had significant difference between visit 1 and visit 5.** The reason maybe the drug still had effect at visit 5, so we could not take 2 cycles into analysis. **So, this analysis was only based on cycle 1.**

Endpoint		XEOMIN	Placebo	Difference	P-value
Change from baseline in DYN_pnch_4_max at week 8 (Change: post-baseline - baseline)	LSMeans (SE)	-3.037(0.629)	0.679(0.5954)	3.716(0.8888)	<0.0001
	95% CI	-4.327(-1.746)	(-0.543,1.901)	1.892(5.539)	
Change from baseline in DYN_pnch_4_Avg at week 8	LSMeans (SE)	-2.323(0.5951)	1.065(0.5688)	3.388(0.848)	<0.0001
	95% CI	-3.544(-1.102)	(-0.102,2.232)	1.648(5.128)	
Change from baseline in DYN_pnch_3_max at week 8	LSMeans (SE)	-2.905(0.7522)	0.557(0.7079)	3.463(1.0336)	0.002
	95% CI	-4.449(-1.362)	(-0.895,2.01)	1.342(5.584)	
Change from baseline in DYN_pnch_3_Avg at week 8	LSMeans (SE)	-2.2(0.6762)	1.144(0.6354)	3.344(0.9303)	0.001
	95% CI	-3.588(-0.813)	(-0.16,2.447)	1.435(5.253)	
Change from baseline in Grip_max at week 8	LSMeans (SE)	-25.04(4.9933)	3.084(4.6889)	28.124(6.9402)	<0.0001
	95% CI	-35.286(-14.795)	(-6.537,12.704)	13.884(42.364)	
Change from baseline in Grip_avg at week 8	LSMeans (SE)	-22.172(4.9974)	4.944(4.721)	27.115(7.0065)	0.001
	95% CI	-32.426(-11.918)	(-4.743,14.63)	12.739(41.491)	
Change from baseline in FF D2-3 max at week 8	LSMeans (SE)	-4.423(6.5695)	19.526(7.1284)	23.949(9.9787)	0.025
	95% CI	-18.013(9.167)	(4.779,34.272)	3.306(44.591)	

Table 8: Primary Efficacy endpoint: MRC scale at the eight-week mark in the active arm compared to the baseline rating.

Method 1: Assumed that the injection effect is no more than 8 weeks, so the visit 1(day 0) and visit 5(week 12, the start of 2nd injection cycle) were considered baseline visit. Note: There were 19 pairs (left and right digit 2,3,4,5) of muscle strength based on raw data for MRC scale, most of scores were 5, so it is hardly find the difference.

Endpoint		XEOMIN	Placebo	Difference	P-value
Change from baseline in	LSMeans	-0.087(0.0654)	0.07(0.0655)	0.157(0.0723)	0.038

R_FDP_dig_2 at week 8 (Change: post-baseline - baseline)	(SE)				
	95% CI	-0.22(0.047)	(-0.063,0.204)	0.009(0.304)	
Change from baseline in R_Lumb_dig_4 at week 8	LSMeans (SE)	-0.119(0.0492)	-0.013(0.0493)	0.106(0.0509)	0.045
	95% CI	-0.219(-0.019)	(-0.113,0.087)	0.003(0.21)	

Method 2: Only cycle 1 was included in the analysis.
No significant change could be found.

Table 9：Secondary endpoint VAS：
Two patients ("MD103-D-M" "MD107-J-E") with outliers, could you please double check to let me know whether the drug had latter effect for them, or they were data issues?
Take "MD107-J-E" as an example, took active drug in the first cycle and placebo in the second cycle, while there was no change in the first cycle, there was significant reduction in the second cycle (week 20). **If these two patients were excluded from analysis, there was significant reduction in VAS score in the active group compared to placebo group.**

For the AP group (took active injection in cycle 1), after comparing the difference between visit 1 (day 0) and visit 5(week 12, the start of 2nd injection cycle) for VAS scale **via paired t-test or paired Wilcoxon sign test** (if normal assumptions are significantly violated), we could not find the significant difference between visit 1 and visit 5.

Method 1: Assumed that the injection effect is no more than 8 weeks, so the visit 1(day 0) and visit 5(week 12, the start of 2nd injection cycle) were considered baseline visit. Note: The analysis result was based on the data in which Two patients ("MD103-D-M" "MD107-J-E") with outliers were removed.

Endpoint		XEOMIN	Placebo	Difference	P-value
Change from baseline in VAS at week 8	LSMeans (SE)	-20.40 (6.47)	-11.33 (6.47)	9.07 (5.83)	0.1295
	95% CI	(-33.53, -7.25)	(-24.50, 1.84)	(-2.80,20.93)	

Method 2: Only cycle 1 was included in the analysis.

Endpoint		XEOMIN	Placebo	Difference	P-value
Change from baseline in VAS at week 8	LSMeans (SE)	-29.77 (7.22)	-10.7 (7.01)	19.07 (10.05)	0.07
	95% CI	(-44.44, -15.11)	(-24.94, 3.54)	(-1.36,39.50)	

(Change: post-baseline - baseline)

Table 10: Secondary Efficacy Endpoint ModHAD：
Scoring the HADS:

- The “A” components are the “anxiety” components.
- The “D” components are the “depression” components.
- Total score 0-7 = normal; 8-10 = borderline; 11-21 = abnormal

For the AP group (took active injection in cycle 1), after comparing the difference between visit 1 (day 0) and visit 5(week 12, the start of 2nd injection cycle) for ModHAD (both A and D components) **via paired t-test or paired Wilcoxon sign test** (if normal assumptions are significantly violated), we could not find the significant difference between visit 1 and visit 5.

Method 1: Assumed that the injection effect is no more than 8 weeks, so the visit 1(day 0) and visit 5(week 12, the start of 2nd injection cycle) were considered baseline visit.

Endpoint		XEOMIN	Placebo	Difference	P-value
Change from baseline in ModHAD at week 8 (A components)	LSMeans (SE)	-2.426 (0.718)	-2.111 (0.7066)	0.315 (0.4917)	0.527
	95% CI	(-3.897, -0.955)	(-3.558, -0.664)	(-0.692,1.3222)	
Change from baseline in ModHAD at week 8 (D components)	LSMeans (SE)	-1.799 (1.038)	-1.561(1.041)	0.237 (0.4608)	0.61
	95% CI	(-3.914, 0.316)	(-3.682, -0.56)	(-0.701,1.176)	

(Change: post-baseline - baseline)

Method 2: Only cycle 1 was included in the analysis.

Endpoint		XEOMIN	Placebo	Difference	P-value
Change from baseline in ModHAD at week 8 (A components)	LSMeans (SE)	-3.533 (0.970)	-3.068 (0.8571)	0.465 (1.2966)	0.7219
	95% CI	(-5.501, -1.565)	(-4.808, -1.328)	(-2.167,3.097)	
Change from baseline in ModHAD at week 8 (D components)	LSMeans (SE)	-1.971 (0.708)	-0.705(0.652)	1.266 (0.966)	0.198
	95% CI	(-3.405, -0.537)	(-2.025, 0.615)	(-0.691,3.224)	

Table 11: Secondary Efficacy Endpoint PSS Perceived Stress Scale

Individual scores on the PSS can range from 0 to 40 with higher scores indicating higher perceived stress.

►Scores ranging from 0-13 would be considered low stress.
►Scores ranging from 14-26 would be considered moderate stress.
►Scores ranging from 27-40 would be considered high perceived stress.

For the AP group (took active injection in cycle 1), after comparing the difference between visit 1 (day 0) and visit 5(week 12, the start of 2nd injection cycle) for PSS **via paired t-test or paired Wilcoxon sign test** (if normal assumptions are significantly violated), we could not find the significant difference between visit 1 and visit 5.

Method 1: Assumed that the injection effect is no more than 8 weeks, so the visit 1(day 0) and visit 5(week 12, the start of 2nd injection cycle) were considered baseline visit.

Endpoint		XEOMIN	Placebo	Difference	P-value
Change from baseline in PSS at week 8	LSMeans (SE)	-0.399 (0.502)	-1.578 (0.5147)	-1.179 (0.5527)	0.041
	95% CI	(-1.4215, 0.6234)	(-2.6259, -0.5292)	(-2.3043, -0.0527)	

Method 2: Only cycle 1 was included in the analysis.

Endpoint		XEOMIN	Placebo	Difference	P-value
Change from baseline in PSS at week 8	LSMeans (SE)	-0.5031(0.946)	-1.429 (0.864)	-0.926 (1.2833)	0.475
	95% CI	(-3.179, 0.3203)	(-3.179, -0.3203)	(-3.5266,1.674)	

Summary of PSS by visit

visitn	avalc	PA	AP	PA_CHG	AP_CHG
1	N	11	10		
1	MEAN (SD)	20.73 (5.985)	18.90 (7.637)		
1	MEDIAN	23.0	20.5		
1	MIN - MAX	6 - 26	1 - 29		
2	N	11	10	11	10
2	MEAN (SD)	19.55 (5.628)	18.40 (7.763)	-1.18 (3.093)	-0.50 (2.718)
2	MEDIAN	21.0	20.0	-1.0	0.0
2	MIN - MAX	5 - 26	0 - 31	-6 - 5	-5 - 3
3	N	11	10	11	10
3	MEAN (SD)	18.64 (5.316)	18.40 (6.552)	-2.09 (2.427)	-0.50 (3.472)
3	MEDIAN	19.0	20.5	-1.0	0.0
3	MIN - MAX	6 - 25	1 - 25	-5 - 2	-6 - 5
4	N	11	9	11	9
4	MEAN (SD)	19.18 (6.431)	18.89 (7.149)	-1.55 (2.841)	-0.56 (3.358)
4	MEDIAN	19.0	19.0	-2.0	0.0
4	MIN - MAX	4 - 30	2 - 26	-6 - 4	-6 - 6
5	N	10	8	10	8
5	MEAN (SD)	18.20 (5.903)	18.38 (6.589)	0.00 (0.000)	0.00 (0.000)
5	MEDIAN	19.0	20.5	0.0	0.0
5	MIN - MAX	4 - 25	3 - 23	0 - 0	0 - 0
6	N	10	8	10	8
6	MEAN (SD)	18.30 (5.579)	17.38 (6.760)	0.10 (2.846)	-1.00 (1.927)
6	MEDIAN	19.0	19.5	0.0	-1.5
6	MIN - MAX	5 - 25	1 - 22	-3 - 6	-4 - 2
7	N	10	8	10	8
7	MEAN (SD)	18.90 (5.705)	18.25 (7.363)	0.70 (2.406)	-0.13 (3.682)
7	MEDIAN	20.0	20.5	0.5	-0.5
7	MIN - MAX	5 - 25	1 - 24	-3 - 5	-6 - 6
8	N	10	7	10	7
8	MEAN (SD)	17.90 (5.666)	15.86 (6.466)	-0.30 (1.767)	-2.14 (2.193)
8	MEDIAN	18.5	19.0	0.0	-2.0
8	MIN - MAX	4 - 25	2 - 20	-2 - 4	-6 - 1
9	N	10	8	10	8
9	MEAN (SD)	17.50 (4.035)	19.88 (3.044)	-0.70 (2.406)	1.50 (6.000)
9	MEDIAN	17.5	20.0	-1.0	-0.5
9	MIN - MAX	8 - 24	16 - 24	-4 - 4	-6 - 14

Note: Cycle1, visitn=1: Day 0; visitn=2: Week 2; visitn=3: Week 4; visitn=4: Week8.
Cycle2, visitn=5: Week 12; visitn=6: Week 14; visitn=7: Week 16; visitn=8: Week20; visitn=9: Final visit Week 24
AP: Received the active drug injection in cycle 1 and placebo in cycle 2. PA: Received the placebo in cycle 1 and active drug injection in cycle 2.

Table 7: Primary Efficacy endpoint: Dynamometer measures at the eight-week mark in the active arm compared to the baseline rating.

Method 1: Assumed that the injection effect is no more than 8 weeks, so the visit 1(day 0) and visit 5(week 12, the start of 2nd injection cycle) were considered baseline visit. Note: There were 34 dynamometer measures, among the 21 patients, only 16 patients had available rating data.
There was significant reduction from baseline rating for 11 measures in active arm compared to placebo group.

Endpoint		Inco-BoNT-A	Placebo	Difference	P-value
Change from baseline in DYN_pnch_5_Avg at week 8 (Change: post-baseline - baseline)	LSMeans (SE)	-0.235(0.4102)	0.549(0.4111)	0.784(0.3675)	0.044
	95% CI	-1.084(0.613)	(-0.302,1.399)	0.024(1.544)	
Change from baseline in DYN_pnch_4_max at week 8	LSMeans (SE)	-1.948(0.5087)	0.536(0.5118)	2.484(0.5831)	<0.0001
	95% CI	-3(-0.895)	(-0.523,1.595)	1.278(3.69)	
Change from baseline in DYN_pnch_4_Avg at week 8	LSMeans (SE)	-1.415(0.4771)	0.648(0.4797)	2.063(0.5132)	0.001
	95% CI	-2.402(-0.428)	(-0.345,1.64)	1.001(3.124)	
Change from baseline in DYN_pnch_3_max at week 8	LSMeans (SE)	-1.767(0.6536)	0.793(0.6551)	2.56(0.68)	0.001
	95% CI	-3.119(-0.415)	(-0.562,2.148)	1.154(3.967)	
Change from baseline in DYN_pnch_3_Avg at week 8	LSMeans (SE)	-1.377(0.5814)	1.17(0.5824)	2.547(0.6298)	0.001
	95% CI	-2.579(-0.174)	(-0.034,2.375)	1.244(3.85)	
Change from baseline in DYN_pnch_2_Avg at week 8	LSMeans (SE)	-1.596(0.691)	0.371(0.6935)	1.966(0.8675)	0.033
	95% CI	-3.025(-0.166)	(-1.064,1.805)	0.172(3.761)	
Change from baseline in Grip_max at week 8	LSMeans (SE)	-16.153(5.0562)	2.93(5.0709)	19.083(4.5297)	<0.0001
	95% CI	-26.613(-5.694)	(-7.561,13.42)	9.712(28.453)	
Change from baseline in Grip_avg at week 8	LSMeans (SE)	-14.699(4.9926)	2.681(5.007)	17.381(5.5336)	0.005
	95% CI	-25.027(-4.371)	(-7.676,13.039)	5.934(28.828)	
Change from baseline in FF D2-3 max at week 8	LSMeans (SE)	-2.737(3.1312)	8.78(3.3806)	11.517(4.5418)	0.019
	95% CI	-9.249(3.774)	(1.75,15.81)	2.072(20.963)	
Change from baseline in pronation maximum pounds of force at week 8	LSMeans (SE)	-1.502(0.7331)	1.046(0.7328)	2.547(0.831)	0.005
	95% CI	-3.018(0.015)	(-0.47,2.562)	0.828(4.267)	
Change from baseline in pronation average pounds of force of 8 tries at week 8	LSMeans (SE)	-1.481(0.67)	1.198(0.6692)	2.679(0.7462)	0.002
	95% CI	-2.867(-0.095)	(-0.187,2.582)	1.135(4.222)	

Method 2: For the A→P group (took XEOMIN injection in cycle 1), after comparing the difference between visit 1 (day 0) and visit 5(week 12, the start of 2nd injection cycle) for all measures **via paired t-test or paired Wilcoxon sign test** (if normal assumptions are significantly violated), **there were 7 measures (DYN_pnch_4_max, DYN_pnch_4_avg, DYN_pnch_3_max, DYN_pnch_3_avg, DYN_pnch_2_max, DYN_pnch_2_avg, Elbox_Flex_avg) had significant difference between visit 1**

and visit 5. The reason maybe the drug still had effect at visit 5, so we could not take 2 cycles into analysis. **So, this analysis was only based on cycle 1.**

Endpoint		Inco-BoNT-A	Placebo	Difference	P-value
Change from baseline in DYN_pnch_4_max at week 8 (Change: post-baseline - baseline)	LSMeans (SE)	-3.037(0.629)	0.679(0.5954)	3.716(0.8888)	<0.0001
	95% CI	-4.327(-1.746)	(-0.543,1.901)	1.892(5.539)	
Change from baseline in DYN_pnch_4_Avg at week 8	LSMeans (SE)	-2.323(0.5951)	1.065(0.5688)	3.388(0.848)	<0.0001
	95% CI	-3.544(-1.102)	(-0.102,2.232)	1.648(5.128)	
Change from baseline in DYN_pnch_3_max at week 8	LSMeans (SE)	-2.905(0.7522)	0.557(0.7079)	3.463(1.0336)	0.002
	95% CI	-4.449(-1.362)	(-0.895,2.01)	1.342(5.584)	
Change from baseline in DYN_pnch_3_Avg at week 8	LSMeans (SE)	-2.2(0.6762)	1.144(0.6354)	3.344(0.9303)	0.001
	95% CI	-3.588(-0.813)	(-0.16,2.447)	1.435(5.253)	
Change from baseline in Grip_max at week 8	LSMeans (SE)	-25.04(4.9933)	3.084(4.6889)	28.124(6.9402)	<0.0001
	95% CI	-35.286(-14.795)	(-6.537,12.704)	13.884(42.364)	
Change from baseline in Grip_avg at week 8	LSMeans (SE)	-22.172(4.9974)	4.944(4.721)	27.115(7.0065)	0.001
	95% CI	-32.426(-11.918)	(-4.743,14.63)	12.739(41.491)	
Change from baseline in FF D2-3 max at week 8	LSMeans (SE)	-4.423(6.5695)	19.526(7.1284)	23.949(9.9787)	0.025
	95% CI	-18.013(9.167)	(4.779,34.272)	3.306(44.591)	

Table 8: Primary Efficacy endpoint: MRC scale at the eight-week mark in the active arm compared to the baseline rating.

Method 1: Assumed that the injection effect is no more than 8 weeks, so the visit 1(day 0) and visit 5(week 12, the start of 2nd injection cycle) were considered baseline visit. Note: There were 19 pairs (left and right digit 2,3,4,5) of muscle strength based on raw data for MRC scale, most of scores were 5, so it is hardly finding the difference.

Endpoint		Inco-BoNT-A	Placebo	Difference	P-value
Change from baseline in R_FDP_dig_2 at week 8 (Change: post-baseline - baseline)	LSMeans (SE)	-0.087(0.0654)	0.07(0.0655)	0.157(0.0723)	0.038
	95% CI	-0.22(0.047)	(-0.063,0.204)	0.009(0.304)	
Change from baseline in R_Lumb_dig_4 at week 8	LSMeans (SE)	-0.119(0.0492)	-0.013(0.0493)	0.106(0.0509)	0.045
	95% CI	-0.219(-0.019)	(-0.113,0.087)	0.003(0.21)	

Method 2: Only cycle 1 was included in the analysis.
No significant change could be found.

Table 9: Secondary endpoint VAS :

For the A→P group (took active injection in cycle 1), after comparing the difference between visit 1 (day 0) and visit 5 (week 12, the start of 2nd injection cycle) for VAS scale **via paired t-test or paired Wilcoxon sign test** (if normal assumptions are significantly violated), we could not find a significant difference between visit 1 and visit 5.

Method 1: Assumed that the injection effect is no more than 8 weeks, so the visit 1(day 0) and visit 5(week 12, the start of 2nd injection cycle) were considered baseline visit. Note: The analysis result was based on the data in which Two patients ("MD103-D-M" "MD107-J-E") with outliers were removed.

Endpoint		Inco-BoNT-A	Placebo	Difference	P-value
Change from baseline in VAS at week 8	LSMeans (SE)	-13.045 (11.426)	1.393 (11.438)	14.439 (3.1357)	<0.0001
	95% CI	(-36.491, 10.398)	(-22.075, 24.861)	(8.0059,20.8738)	

Method 2: Only cycle 1 was included in the analysis.

Endpoint		Inco-BoNT-A	Placebo	Difference	P-value
Change from baseline in VAS at week 8	LSMeans (SE)	-19.753 (7.10)	-6.710 (6.947)	13.043 (9.933)	0.198
	95% CI	(-34.174, -5.332)	(-20.829, 7.409)	(-7.142,33.229)	

(Change: post-baseline - baseline)

Table 10: Secondary Efficacy Endpoint ModHAD：
Scoring the HADS:

- The "A" components are the "anxiety" components.
- The "D" components are the "depression" components.
- Total score 0-7 = normal; 8-10 = borderline; 11-21 = abnormal

For the A$\rightarrow$P group (took active injection in cycle 1), after comparing the difference between visit 1 (day 0) and visit 5(week 12, the start of 2nd injection cycle) for ModHAD (both A and D components) **via paired t-test or paired Wilcoxon sign test** (if normal assumptions are significantly violated), we could not find the significant difference between visit 1 and visit 5.

Method 1: Assumed that the injection effect is no more than 8 weeks, so the visit 1(day 0) and visit 5(week 12, the start of 2nd injection cycle) were considered baseline visit.

Endpoint		Inco-BoNT-A	Placebo	Difference	P-value
Change from baseline in ModHAD at week 8 (A components)	LSMeans (SE)	-2.426 (0.718)	-2.111 (0.7066)	0.315 (0.4917)	0.527
	95% CI	(-3.897, -0.955)	(-3.558, -0.664)	(-0.692,1.3222)	
Change from baseline in ModHAD at week 8 (D components)	LSMeans (SE)	-1.799 (1.038)	-1.561(1.041)	0.237 (0.4608)	0.61
	95% CI	(-3.914, 0.316)	(-3.682, -0.56)	(-0.701,1.176)	

(Change: post-baseline - baseline)

Method 2: Only cycle 1 was included in the analysis.

Endpoint		Inco-BoNT-A	Placebo	Difference	P-value
Change from baseline in ModHAD at week 8 (A components)	LSMeans (SE)	-3.533 (0.970)	-3.068 (0.8571)	0.465 (1.2966)	0.7219
	95% CI	(-5.501, -1.565)	(-4.808, -1.328)	(-2.167,3.097)	
Change from baseline in ModHAD at week 8 (D components)	LSMeans (SE)	-1.971 (0.708)	-0.705(0.652)	1.266 (0.966)	0.198
	95% CI	(-3.405, -0.537)	(-2.025, 0.615)	(-0.691,3.224)	

Table 11: Secondary Efficacy Endpoint PSS**: Perceived Stress Scale**
Individual scores on the PSS can range from 0 to 40 with higher scores indicating higher perceived stress.

- ►Scores ranging from 0-13 would be considered low stress.
- ►Scores ranging from 14-26 would be considered moderate stress.
- ►Scores ranging from 27-40 would be considered high perceived stress.

For the A→P group (took active injection in cycle 1), after comparing the difference between visit 1 (day 0) and visit 5(week 12, the start of 2nd injection cycle) for PSS **via paired t-test or paired Wilcoxon sign test** (if normal assumptions are significantly violated), we could not find the significant difference between visit 1 and visit 5.

Method 1: Assumed that the injection effect is no more than 8 weeks, so the visit 1(day 0) and visit 5(week 12, the start of 2nd injection cycle) were considered baseline visit.

Endpoint		Inco-BoNT-A	Placebo	Difference	P-value
Change from baseline in PSS at week 8	LSMeans (SE)	-0.399 (0.502)	-1.578 (0.5147)	-1.179 (0.5527)	0.041
	95% CI	(-1.4215, 0.6234)	(-2.6259, -0.5292)	(-2.3043, -0.0527)	

Method 2: Only cycle 1 was included in the analysis.

Endpoint		Inco-BoNT-A	Placebo	Difference	P-value
Change from baseline in PSS at week 8	LSMeans (SE)	-0.5031(0.946)	-1.429 (0.864)	-0.926 (1.2833)	0.475
	95% CI	(-3.179, 0.3203)	(-3.179, -0.3203)	(-3.5266,1.674)	
